# Physico-chemical characterization of *Antheraea mylitta* silk mats for wound healing applications

**DOI:** 10.1038/s41598-017-10531-7

**Published:** 2017-09-04

**Authors:** G. H. Darshan, Dexu Kong, Julien Gautrot, Shyamkumar Vootla

**Affiliations:** 1 Department of Biotechnology and Microbiology, Karnatak University, Dharwad, 580 003 Karnataka India; 2 School of engineering and Material Science, Queen Mary University of London, Mile End Road, London, E1 4NS UK

## Abstract

In the field of plastic reconstructive surgery, development of new innovative matrices for skin repair is in demand. The ideal biomaterial should promote attachment, proliferation and growth of cells. Additionally, it should degrade in an appropriate time period without releasing harmful substances, not exerting a pathological immune response. The materials used should display optimized mechanical properties to sustain cell growth and limit scaffold contraction. Wound healing is a biological process directed towards restoration of tissue that has suffered an injury. An important phase of wound healing is the generation of a basal epithelium wholly replacing the epidermis of the wound. Wild silk from *Antheraea mylitta* meets these demands to a large extent. To evaluate the effects of the treatment, *Antheraea mylitta* and *Bombyx mori* samples were characterized by SEM-EDX, FT-IR, XRD and TGA-DSC techniques. Preliminary cell growth behavior was carried out by culturing epidermal cells and proliferation was quantified via viability assay. Moreover, *Antheraea mylitta* possesses excellent cell-adhesive capability, effectively promoting cell attachment and proliferation. *Antheraea mylitta* serves as a delivery vehicle for cells. With all these unique features, it is expected that *Antheraea mylitta* mat will have wide utility in the areas of tissue engineering and regenerative medicine.

## Introduction

One goal in the field of biomaterials is to fabricate matrices that mimic the structure and biological function of the extracellular matrix (ECM)^1^. ECM is made out of two principle types of macromolecules, glycosaminoglycans and proteins such as collagen and fibronectin, which together shape a dense mesh of fibres scaffolding tissues. For example, collagen fibres organize into a three-dimensional matrix structured at multiple length scales to confer robust mechanical properties to the ECM as well as promote cell adhesion^2, 3^. Similarly, the scaffolds designed for tissue regeneration and wound healing need to accomplish similar functions (mechanical structure and promoting cell adhesion) in order to guide cell phenotypes such as proliferation and differentiation. Furthermore, biocompatibility and biodegradability, with non-toxic and non-inflammatory degradation products during replacement *in vivo* by cellular ECM components are key criteria^4^.

Natural and synthetic polymers and a hybrid of both have been used widely for the design of biocompatible materials for applications as skin grafts. Commercialized skin grafts are mainly constructed from collagen and biodegradable polymers, such as poly (ε-caprolactone) (PCL)^5^, poly (lactic acid) (PLA)^6^, poly(lactic-co-glycolic acid) (PLGA)^7^, and poly (glycolic acid) (PGA)^8^, can be manufactured reproductively on a large scale, and can also be processed into biocompatible material in a more controllable manner. The materials macro/micro structure, mechanical properties, and degradation rate can be relatively easily tuned and manipulated to address the requirements of a range of different applications^9, 10^.

A major drawback of synthetic polymer scaffolds is that they are not biologically active, in contrast, to naturally occurring ECM (extracellular matrix): they do not have specific motifs on their surface for specific cell targeting and binding, nor do they provide soluble factors for correct cell growth and development. RGD-containing peptide sequences and many other cell adhesive peptides, saccharides, and proteins have been successfully conjugated to the surface of biomaterials using adsorption or covalent grafting methods, in order to confer bioactivity to the polymer and promote cell adhesion. Enhanced cell adhesion and improved cell proliferation have been observed through these treatments^11–13^.

Several research groups have focused on electrospinning for the design of fibrous matrices, with substantial progress. One of the disadvantages of the degumming procedure is that it results in detrimental changes in the mechanical properties of silk fibroin^14^. Furthermore, the extent to which these properties are affected may depend upon the method used^15^. Because of the complex and not yet fully characterized construction electrospinning silk, the design of artificial fibres with similar properties to native dragline silk proves to be still a complex challenge^16, 17^. An ideal biomaterial should be non-toxic, non-immunogenic and sustain cell adhesion, migration, proliferation and remodelling of the wound bed, in order to promote tissue regeneration^18, 19^.

Silkworm silk has long been used in the textile industry as well as for sutures in medical applications. In the last few decades, silk has also been exploited as a potential biomaterial in tissue engineering applications for its various desirable properties such as cell attachment, cytocompatibilty, biodegradability, enhanced mechanical and tensile strength, as well as versatile processing options, in order to meet tissue-specific requirements. Silk is composed of two main protein components: A water-soluble protein glue, sericin, that bonds the crystalline fibroin together to form the natural silk fiber. Sericin usually constitutes 20–30% of silk protein in cocoon^20, 21^. Sericin is a group of polypeptides comprised of 17–18 types of amino acids^22, 23^, most of which have strong polar side groups such as hydroxyl, carboxyl, and amino groups with high serine, aspartic acid, and glycine are the three most abundant amino acids^24^. Sericin from non-mulberry cocoons has been investigated for their ability to support the growth of skin cells^25^. Fibroblast cells were grown on cross-linked sericin films which showed enhanced fibroblast attachment and viability. The membranes also exhibited slow degradation and excellent mechanical properties^26, 27^. Thus sericin protein membranes can open up new dimensions for skin tissue repair. Silk sericin protein from *A*. *mylitta* has also been found effective in inhibiting UVB-induced apoptosis in human skin keratinocytes^28^. Sericin becomes immunogenic only when it is associated with fibroin^29^.

Mechanical properties are important structural variants of silk fibre. As, the quality parameters of the mulberry and non-mulberry can be measured with the variation of the tensile properties including strain, tenacity, Young’s modulus, toughness etc. *A*. *mylitta* have favourable properties for tissue engineering/biomaterial offering advantages such as relative ease in fabrication into diverse morphologies, ease of processability, exceptional flexibility and porosity. The fact that *A*. *mylitta* silk is a biocompatible natural protein provides silk materials with added advantages, such as minimal immune response, good adherence and growth of cells on/in silk matrices. The main advantage of the present approach is that it provides a unique opportunity to broaden the application of Indian tasar silkworm *A*. *mylitta* for tissue regeneration.

The chemical structure of *A*. *mylitta* (Am) silk fibroin is remarkably different from that of *B*. *mori* (Bm) silk fibroin. In comparison with the amino acid composition of *B*. *mori* silk fibroin, the alanine residues content of *A*. *mylitta* silk fibroin is higher than that of *B*. *mori* silk fibroin. Additionally, it contains abundant aspartic and arginine which can form the famous sequence: Arg-Gly- Asp (RGD) sequences which favours cell attachment. There are many repeated -(Gly-Ala-Gly-X)n sequences in the crystalline region of *B*. *mori* silk fibroin where X refers to Ser or Tyr^30, 31^.

Much of the work has been accomplished using mulberry silk, produced by *B*. *mori*, a domesticated silkworm and has been studied for use in surgery and tissue engineering, but the use of non-mulberry silk, *A*. *mylitta* has not received much attention, despite its higher mineralization content. An important hurdle in this respect is the development of methodologies allowing the deposition of silk mats of *A. mylitta*, more suitable for tissue engineering applications than cocoons. This study presents a simple methodology enabling the deposition of silk mats of *A*. *mylitta* and the study of the physico-chemical as well as surface properties of the *A*. *mylitta* and *B*. *mori* silks, with the aim to determine the suitability of wild silk mats as a biocompatible potential substratum for supporting cell adhesion and proliferation.

## Results and Discussion

We examined *A*. *mylitta* and *B*. *mori* silk mat in *in vitro* culture systems to determine whether this material supported the growth of human immortalized keratinocyte (HaCaTs) cells. The silk mats were prepared for culture with cells by cutting mats into 1 cm × 1 cm pieces^32^.

### Surface morphology of Silk mats

**Figure 1:**
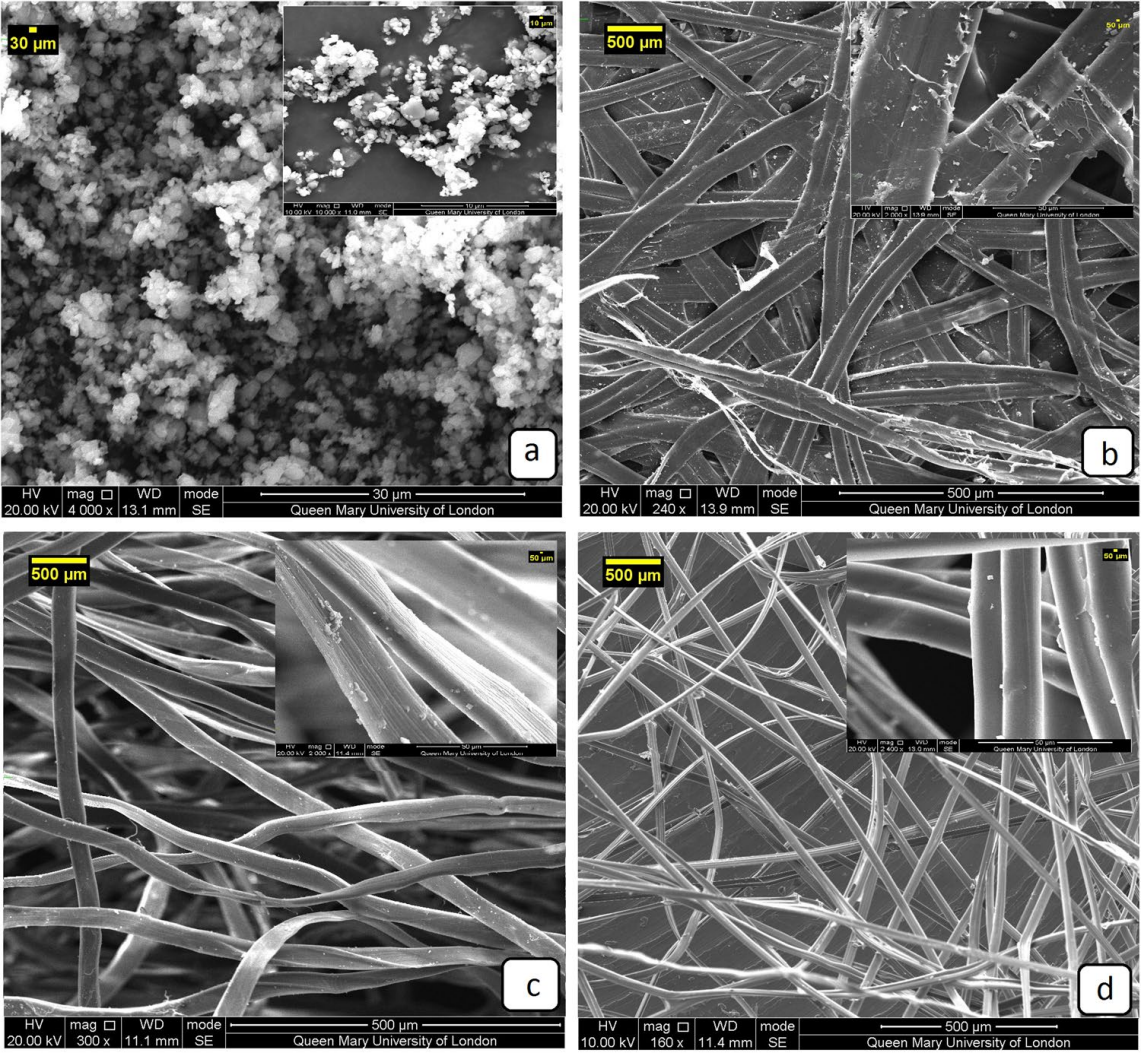
Scanning electron microscopy of the materials (**a**) calcium oxalate monohydrate (**b**) Am, (**c**) Degummed Am and (**d**) Bm.

The silk mats outer surface Fig. 1b–d illustrates the wide range of structures evolved by the Lepidopteran larva. Figure 1a depicts surface morphology of pure calcium oxalate monohydrate. At the most general level, both of the mats have a connected and porous fibre structure. Bm mats have a highly porous non-woven structure, while Am mats have very low porosity with the fibres densely packed and bonded by an almost continuous film of sericin. Mats of Am had a strong silk thread (peduncle) spun to attach the cocoon to the twig of a plant which is necessary during cocoon spinning^33^. The peduncle was composed of highly cross-linked fibers embedded with a mass of sericin gum. Calcium oxalate crystals were found on Am (Fig. 1b) surfaces^34^, and this feature may have a functionality role such as preferential gating of CO_2_ from cocoon inside to outside and temperature regulation to maintain a physiological temperature inside the cocoon irrespective of the surrounding environment^35^. The attachment surfaces of Bm (Fig. 1d) were smooth and plastered with large amounts of gum and crystals while Am cocoon was covered with white rectangular and cubic shaped granular crystalline materials throughout the surface except for areas of fiber intrusion. FTIR spectra showed the peaks at 1632 cm^−1^, 1317 cm^−1^ and 778 cm^−1^ were attributed to calcium oxalate crystal monohydrate. However, Am mats display a prominently higher level of mineralisation than Bm.

SEM images clearly show that the Am cocoon mats are intact on their surface with denser, more packed fibers compared to Bm (Fig. 1b,d). The compactness of Am cocoons can be attributed to the evolution of the corresponding species: Am silkworms undergo pupal diapause which denotes robust construction and compactness of pupa inside the cocoon for 4 months, which is not the case of Bm pupa and associated cocoons. However, the micrographs of samples degummed by alkali method show that extensively degummed fibers have a relatively smooth and clean surface as seen from Fig. 1c, but that all the sericin was not removed from the fibers. The fiber surface shows some evidence of very fine longitudinal striation attributable to the fibrillar structure of the truly degummed silk fibers^36^.

### Composition of Silk mats

**Figure 2:**
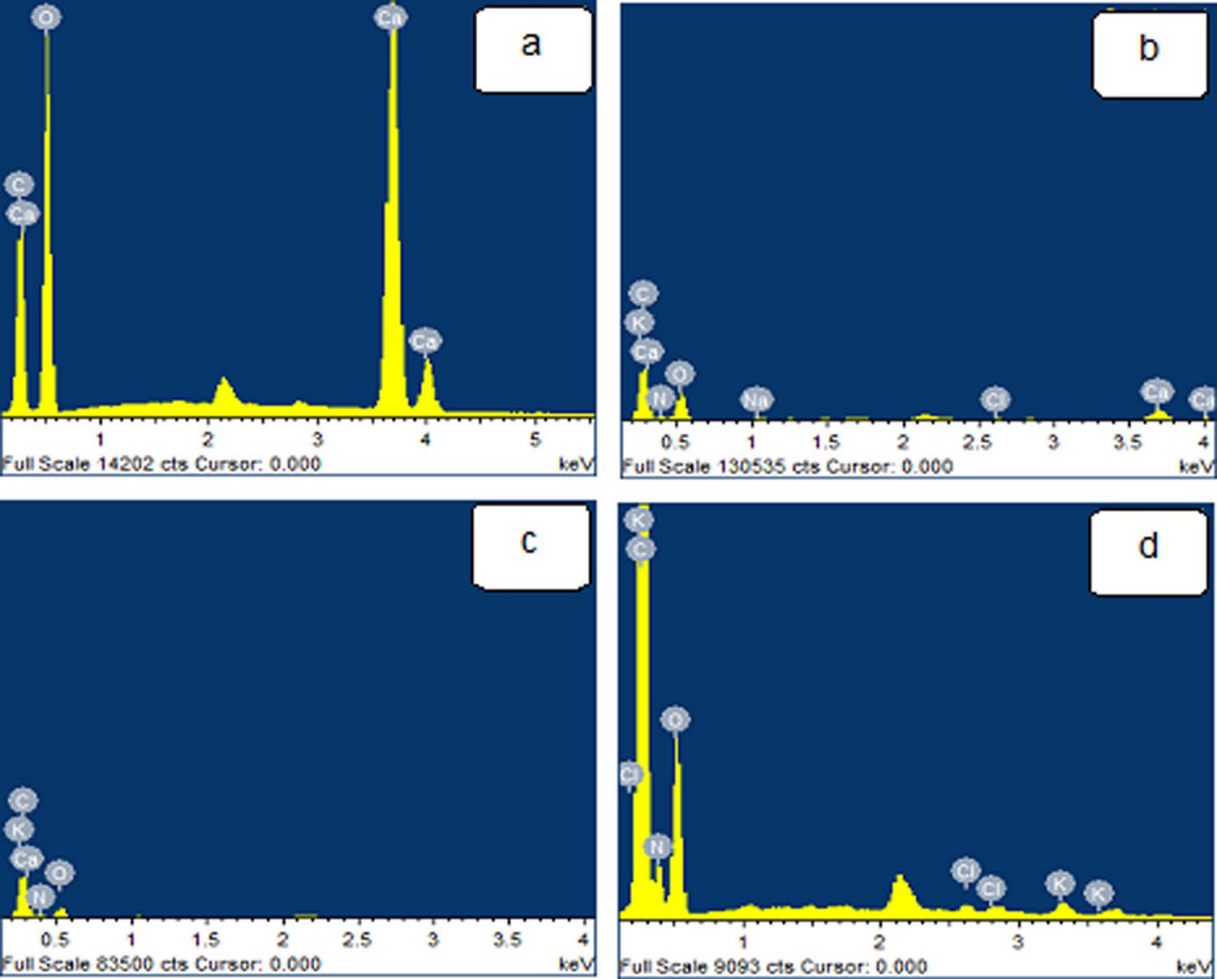
Energy dispersive X-ray spectroscopy (EDX) analysis showing the presence of elements in materials (**a**) Calcium oxalates (**b**) Am (**c**) Degummed Am and (**d**) Bm.

Figure 2 and Table 1 gathers elemental distributions measured for the different samples, using EDX. Apart from carbon (C), Nitrogen (N) and oxygen (O); Am, degummed Am and Bm mats reveal trace amounts of potassium (K) and chloride (Cl) could be detected (Fig. 2b–d). Calcium (Ca) is found in significant concentrations in Am mats (Fig. 2b), whereas it is found in trace amounts in degummed Am mat (Fig. 2c). Dermal excitation and inhibition are associated with the influx of calcium and chloride ions, respectively, into the skin. Activation of excitation receptors in cultured human keratinocytes similarly induces calcium ion influx and topical application of tissue recovery^37^. The highest concentration of calcium in *Antheraea* is understandable since several wild cocoon species have high concentrations of calcium oxalate crystals and trace amount of Zn and P can be detected in Am^38–42^.

**Table 1:** Summary of elemental analysis for different elements (wt%) present in different silk mats.

Samples	C	N	O	Na	Cl	Ca	K
Calcium oxalate	14.79	—	56.52	—	—	28.70	—
Am	43.21	12.36	38.65	0.26	0.42	5.87	0.14
Degummed Am	51.71	20.37	27.30	—	0.20	0.35	0.08
Bm	51.64	19.60	28.44	—	0.36	—	0.57

In spite of the fact that these reviews exhibited the presence of calcium oxalate crystals on the surface however we are revealing that calcium oxalate is surprisingly unevenly dispersed on the surfaces *A*. *mylitta* silk mats compared to *B*. *mori*. Calcium oxalate crystals are thought to affect the thermal behaviour of silkworm cocoons, by keeping the still air trapped inside the cocoon structure and enhancing the thermal stability of the cocoon assembly^35^.

### Structural characteristic of silk mats

**Figure 3:**
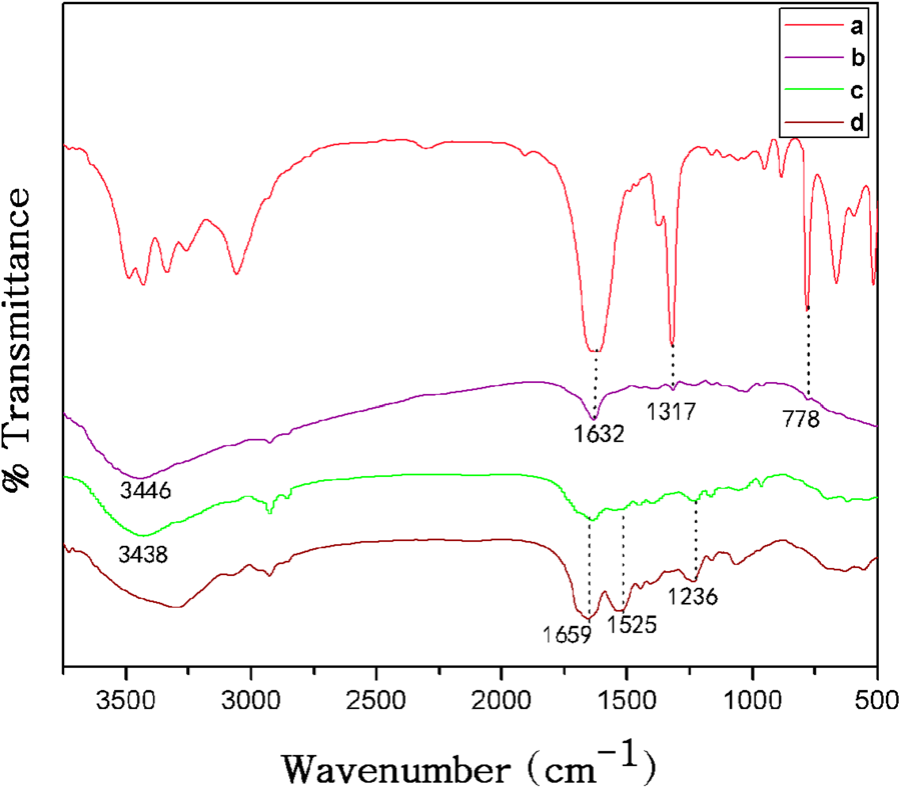
FTIR spectra of (**a**) Calcium oxalates, (**b**) Am (**c**) Degummed Am and (**d**) Bm.

FTIR spectroscopy is useful in the structural analysis of silk because the position and intensity of the amide bonds are sensitive to the molecular conformation and assembly of the corresponding protein chains. In Fig. 3a, the FTIR spectra showed absorption peaks at 1632 cm^−1^, 1317 cm^−1^ and 778 cm^−1^ for the calcium oxalate commercial powder. Whereas strong vibration at 1,632 cm^−1^ is assigned to asymmetric C=O vibration and the strong vibration at 1317 cm^−1^ is assigned as symmetric C=O vibration of the oxalate group^35^. Similarly, all three peaks are present in the Am mat (Fig. 3b). The absence of the peaks at 1632 cm^−1^, 1317 cm^−1^ and at 778 cm^−1^ indicates that the degummed Am and Bm mats do not contain calcium oxalate crystals. Thereby decreasing the cocoon porosity which lessen the mechanism of cocoon tensile properties. The characteristics of the IR spectra of amide I (1700–1600 cm^−1^), amide II (1540–1520 cm^−1^) and amide III (1300–1220 cm^−1^) of silk proteins^43^. Degummed Am (Fig. 3c) and Bm (Fig. 3d) are characterized by the absorption bands at 1659 cm^−1^ (amide I), assigned to random coil conformation, 1525 cm^−1^ (amide II) and 1236 cm^−1^ (amide III), attributed to the β-sheet structure. Similarly, the higher wavenumber broad absorption near 3446 cm^−1^ for Am and 3438 cm^−1^ for degummed Am mats indicates the presence of other functional groups in addition to O-H bonds arising from the presence of water. The weak vibration below 3000 cm^−1^ in all samples is attributed to CH bonds from the silk protein backbone and side chains.

### Physical structure of silk mats

**Figure 4:**
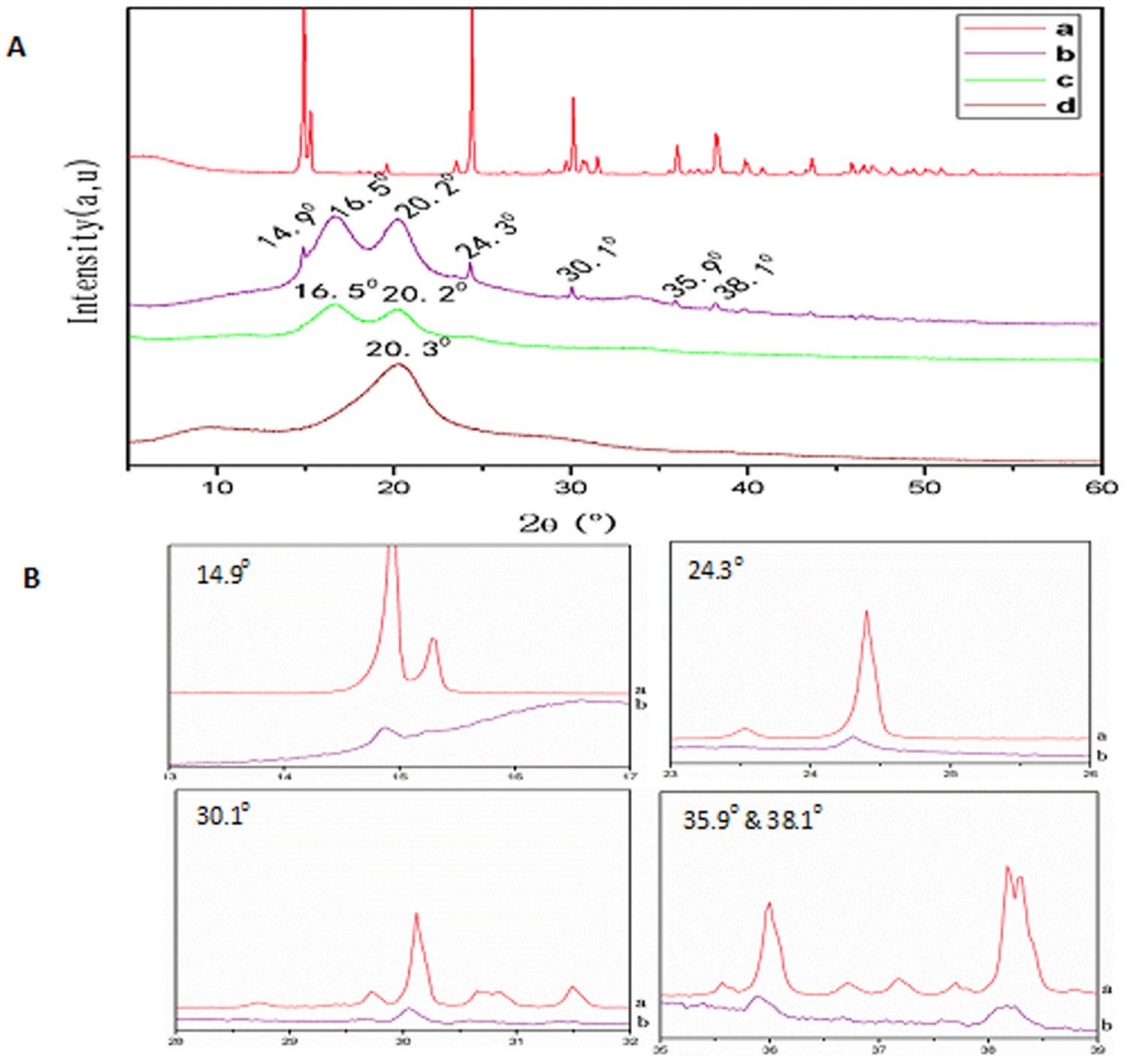
(**A**) The measured X-ray diffraction profile obtained from (a) Calcium oxalates, (b) Am, (c) Degummed Am and (d) Bm. **(B**) X-ray diffractogram of Calcium oxalates compared to Am mat.

X-ray diffraction measurements were carried out to determine the structural changes of Am, degummed Am and Bm mats. The measured temperature ranged from room temperature to 70 °C, and diffraction profiles were collected at every rise of 1 °C and the X-ray diffraction patterns have been determined as follows: 11.8° and 22.0° for α-helix structure, and 16.5°, 20.2°, 24.9°, 30.9°, 34.59°, 40.97° and 44.12° for β-sheet structure^44^. Parts of the measured diffraction profiles are shown in Fig. 4. First we obtained the XRD pattern of pure calcium oxalate monohydrate and Fig. 4a shows the characteristic peaks around at 15°, 24.93°, 30.1°, 35.9°, 38.1°, 39.76° and 43.72°. There is no doubt about the identity of Am mats peaks at 14.9°, 24.93°, 30.1°, 35.9° and 38.1° (Fig. 4b) whose diffraction pattern is superimposable with the XRD pattern of pure calcium oxalate monohydrate. The X-ray diffraction intensity curves of Am reveals two peaks at 16.5° and 20.2° (Fig. 4b), and a single broad peak at 20.2° for Bm (Fig. 4d) are typical of silk fibers. Further, peaks around at 16.5° and 20.2° in XRD curve which indicate much β- sheet structure and high crystallinity when compared to Bm^45^. Silk degradation could be regulated by changing crystallinity and the degradability of silk can be related to the mode of processing and the corresponding content of beta sheet crystallinity. The difference in degradation was due to increased surface roughness or differences in content or distribution of crystallinity^46^. The diffraction patterns remained unchanged regardless of degummed Am (Fig. 4c), confirming that the crystalline structure was not directly affected by the chemical agent and further confirming the nature of the inorganic residues observed at the surface of silk fibers. The differences in diffraction peaks between Am and Bm were attributed to the differences in the unit cell dimensions.

### DSC analysis

**Figure 5:**
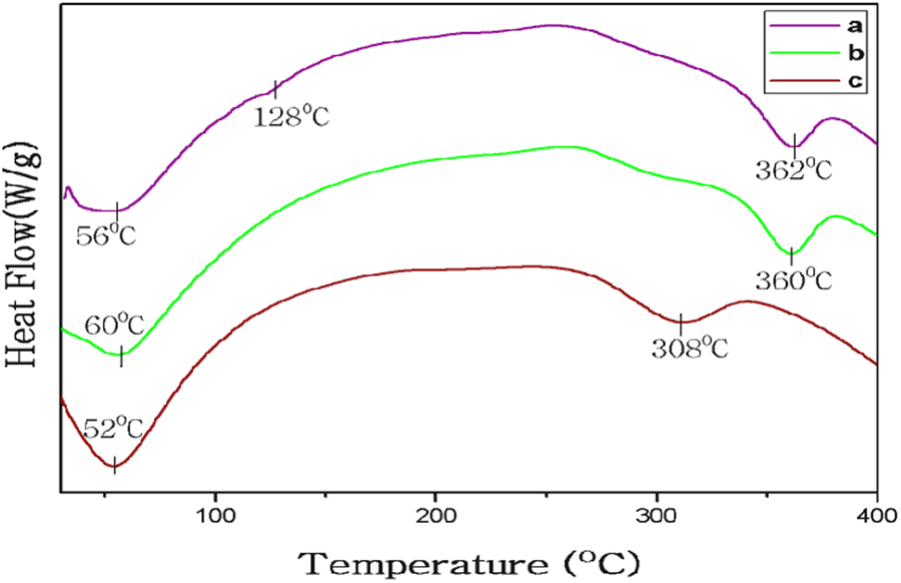
Standard DSC scans of different mats. (**a**) Am (**b**) Degummed Am and (**c**) Bm.

The thermal properties of different silk mats were first examined by standard DSC. Figure 5 depicts the standard DSC scans of Am, degummed Am and Bm mats. During the heating scans, Am mat and degummed Am first showed a peak corresponding to bound water evaporation, near 56 °C and 60 °C respectively. The bound water peaks of Bm mat started from about 40–60 °C^47, 48^. This indicates the silk sericin gums on the surfaces of Am mats may contain looser bound water molecules than that in degummed Am mat. In nature, this advantage could help them exchange water molecules easily with different atmospheres, while the fibers are rigid enough to protect the wild silkworm cocoons in an extremely wild environment^49, 50^. After the water removal, a small endothermic peak appeared at 128 °C for Am sample (Fig. 5a) attributed to a well-oriented β-sheet crystalline conformation, but which is not appearing in degummed mats or Bm mats. Meanwhile, the Bm maintained stable heat flow without any significant change in this region. Again, this highlights major differences between wild Am and domesticated Bm mats. The degradation peak temperatures of Bm mat was significantly lower than Am and degummed Am mats. With the help of heat-protective sericin layer, the Bm mat has a degradation peak around 308 °C (Fig. 5c). In comparison, the Am and degummed Am mats are much higher and are around 362 °C and 360 °C respectively (Fig. 5a and b), indicating that wild silks are more thermally stable than domesticated silks in nature.

### Thermal Behaviour

**Figure 6:**
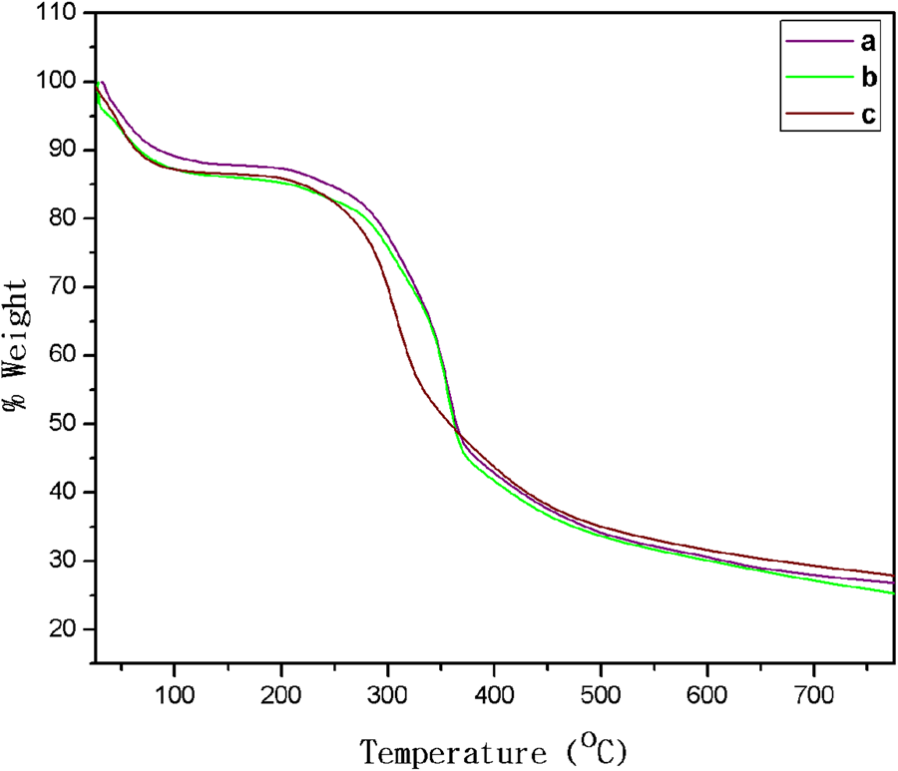
Thermogravimetric curves of (**a**) Am (**b**) Degummed Am and (**c**) Bm.

To quantify the water content and study thermal degradation processes of samples, TGA was performed on the different silk mats. Generally, silk fibers degrade through three main stages: (i) starting from 70 °C, the moisture absorbed during storage will be released from the silk fibers; (ii) a second transition, from 265 °C to 380 °C, the silk fibers undergo an abrupt degradation; and (iii) from 380 °C onwards, the silk fibers start to decompose^51, 52^. Figure 6 reveals the mass percentage change of Am, degummed Am and Bm mats during heating from room temperature to 800 °C. During the initial heating from room temperature to about 100 °C, bound water molecules were removed from all silk samples as we demonstrated in the DSC study. However degummed Am mats still maintained water molecule as compared to Am and Bm mats. Am and degummed Am samples showed stable masses in the temperature region from 80 °C to about 310 °C and Bm from 70 °C to about 270 °C respectively^53^.

Above 314 °C, the major thermal degradation process started for Am and degummed Am samples degraded together between 312 °C–380 °C and their degradation rates slowed down above 664 °C and 626 °C respectively (Fig. 6a and b). After this, Bm samples showed the fastest weight loss 270 °C–356 °C in Fig. 6c as we predicted in the DSC study and degradation rates slowed down above 598 °C. Above this temperature, no peaks were observed in the first derivative and all traces decreased to plateau around 25 weight %. The Am and degummed silk samples, there is a significant difference of remaining mass 32. 5% & 35% at 664 °C and 622 °C respectively and thermal stability of the Am was enhanced by the sericin which acts as barriers for better heat insulation. For the Bm sample, the remaining mass (30%) at 598 °C, suggesting that the thermal stability of Bm cocoons in high-temperature regions are weaker than wild silk samples. Polymorphs of a crystalline structure and amino acid composition of Am are different from those of Bm silk fiber. Therefore, the thermal degradation behaviour is expected to be different between Am and Bm silk^54^.

### Cell Culture

**Figure 7:**
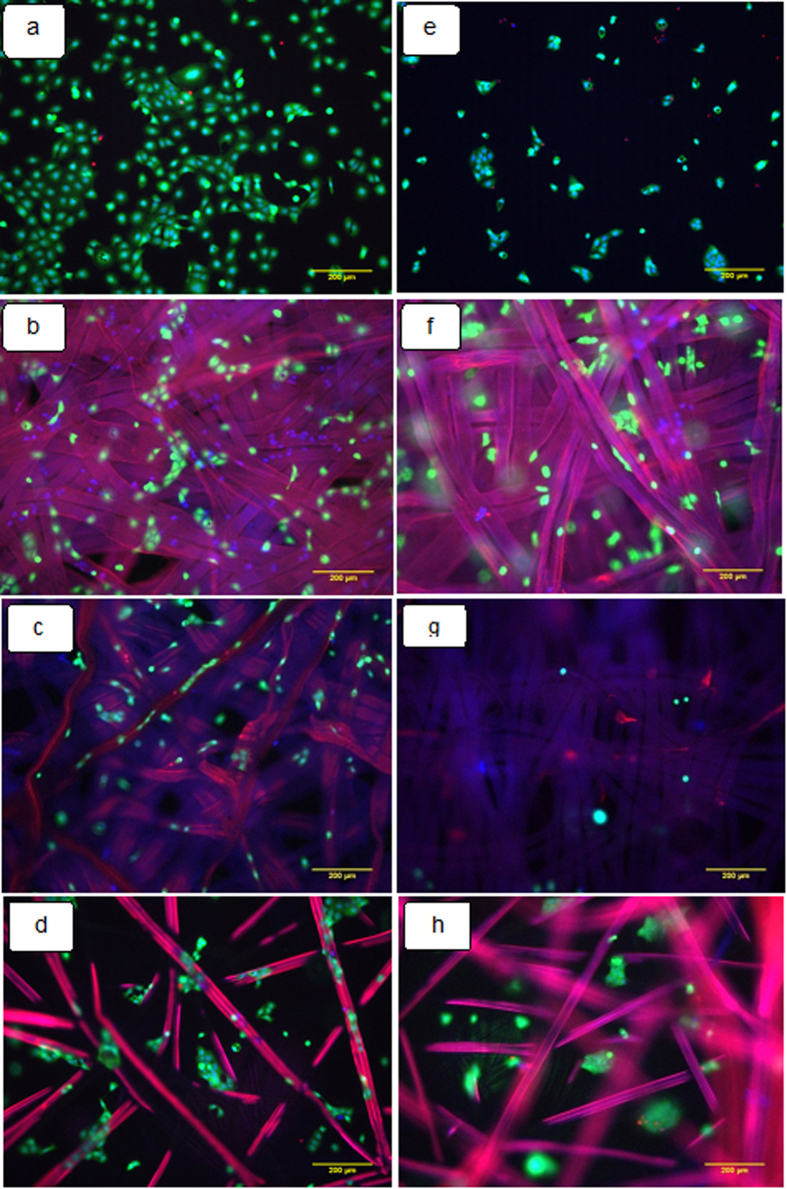
Epi fluorescence microscopy images of keratinocytes seeded on the different substrate after 24 h. Tissue culture plate (**a**,**e**), Am (**b**,**f**), Degummed Am (**c**,**g**) and Bm (**d**,**h**). (**a**–**d**) Samples coated with collagen before cell seeding and (**e**–**h**) no collagen. Scale bars represent 200 μm.

**Figure 8:**
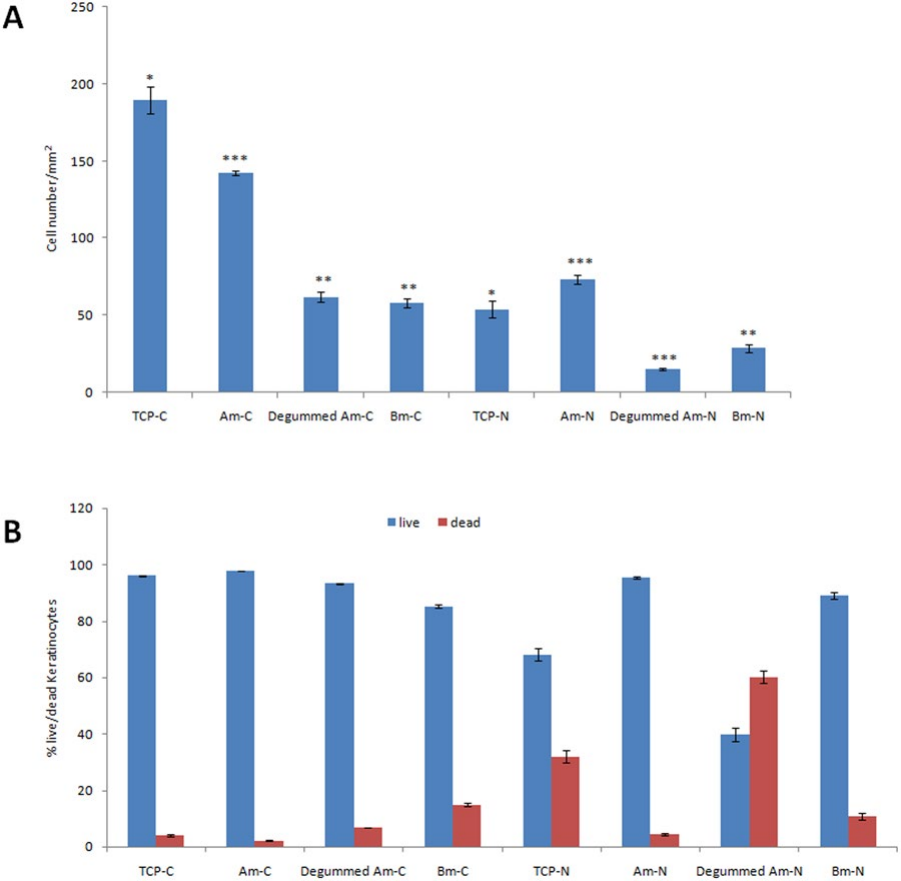
Keratinocytes efficiently attach on different silk mats. **(A)** Quantitative analysis of an adhesion assay of keratinocytes. **(B)** Viability assay of keratinocytes on different silk mats. The mats surface area covered by cells represented as a mean ± standard deviation of three fields studied for each type of mats. (**C**) denotes samples treated with collagen and N represents no collagen. Each point represents mean from three independent experiments. The error bars denote standard deviation, n = 3, *p < 0.05, ***p < 0.01 represent a significant statistical difference between control and different silk. The 2D image analysis was performed with ImageJ software.

Tissue engineering allows the combination of cells with scaffolds or biomaterials in order to regenerate a tissue or organ *in vivo* or *in vitro* ^55^. This process not only requires the growth of relevant cells on the biomaterial but also the growth and spread of a vascular system to support the functioning and maintenance of the regenerated tissue^56^. The cytocompatibility of the materials is essential for their application in wound healing. Since it is the biomaterial surface that first comes into contact with the living tissue when the biomaterial is implanted, the initial response of the body to the biomaterial depends on its surface properties. Surface properties that can influence biocompatibility include surface charge, surface topography and hydrophilicity^57^. In our present study, cell attachment morphology and viability were studied on different silk mats. The cells appeared more spread on tissue culture plate (TCP) and *A*. *mylitta* mat than on degummed Am and *B*. *mori* mat (Fig. 7). The cell spreading on the different mats coated with collagen is comparable to that of tissue culture plastic treated with collagen as shown in Fig. 7. In the absence of collagen, cell spreading appeared restricted on all samples. Figure 8A presents the quantification of cell attachment (cell densities per surface area) and viability (Fig. 8B) on Am, degummed Am and Bm mats. All three silk types were found to be cytocompatible and did not display significant toxicity after coating with collagen. However, cell densities were significantly increased on TCP and Am than on degummed Am and Bm mats. Epi fluorescence microscopy revealed keratinocytes formed flattened cells with very tight cellular contacts both on and between individual fibroin fibers. These cells adhered poorly to native mats but adhered significantly better when the mats were first coated with collagen, consistent with their use of integrins such as α3β1 integrins to anchor to ECM and spread.

The increased adhesion of cells on Am mats may be due to their high abundance in serine residues, their high mineralization level and the high content of sericin typical of these fibres Therefore, in addition to their comparatively rougher surface, such changes in physicochemical properties could make Am mats better substrates for cell attachment and growth than Bm mat. To understand the potential role of the glue-like sericin proteins that coat the non-extracted native Am, the cytocompatibility of pristine and degummed mats were compared. The % of glycine amino acid is high in *A*. *mylitta* cocoons as compared to *Bombyx mori*. Kaori Inoue^58^ reported that glycine is functionally active in human epidermal keratinocytes. We observed that pristine Am mats with sericin sustained the adhesion of higher cell densities whilst preserving excellent cytocompatibility. This occurred even after collagen treatment. This finding suggests that the Am mats and their natural sericin coating are interesting features for promoting the adhesion of keratinocytes, likely as a result of increased ECM protein deposition.

## Conclusion

Wild silk mats display interesting features as a novel biomaterial for application in wound healing and tissue engineering. In particular, their unique dense morphology that mimic the dense basement membrane of epithelial tissues such as in the inter follicular epidermis of the skin, their natural ability to sustain cell adhesion, in the absence of collagen deposition, and their mineralization, which may provide useful cues for the proliferation and differentiation of stem cells. The surface morphology of *Antheraea mylitta* is relatively compact and rough but still displaying some micro porosity, compared to Bm silk mats. The physical properties of silk fibroin are altered by the occurrence of the β-sheet formation. Moreover, pristine *Antheraea mylitta* silk mats promote the adhesion of keratinocytes even in the absence of collagen coating, in contrast to *Bombyx mori* silk mats. Such impact on cell adhesion seems to be mediated by the natural sericin coatings of Am silk mats as this behaviour was not observed on degummed fibers. This study confirms the potential of native cocoons particularly of Indian tropical non-mulberry *Antheraea mylitta* origin as biomaterials for regenerative medicine and tissue engineering. However, interesting challenges remain in the development of their use and fabrication. For example, *Antheraea mylitta* cannot be cultured in similar conditions to *Bombyx mori*, hence requiring the development of novel culture protocols for non-domesticated silk worms, or the genetic engineering of *Bombyx mori* to promote the formation of higher contents of sericin and accumulation of mineral components conferring unique physical and cytocompatibility properties to wild silks.

## Methods

### Materials

Dulbecco’s Modified Eagle’s Medium, Trypsin and Versene (Thermofisher Scientific), Fetal Bovine Serum (FBS, Labtech), L-Glutamine and Penicillin-Streptomycin (Invitrogen), Collagen type I (Corning), Calcein AM, Ethidium homodimer, Hoechst 33342 (Thermo fisher Scientific) were purchased for this experimentation. Sodium Carbonate (Na_2_CO_3_) was purchased from Sigma-Aldrich.

Tasar silk cocoons produced by *Antheraea mylitta* (Am) were procured from Warangal district in Telangana, India and Mulberry cocoons by *Bombyx mori* (Bm) silkworms were reared in our laboratory room at 22 ± 3 °C and 68 ± 7% relative humidity and fed with mulberry leaves until they spun cocoons. The cocoons were cut to separate the pupae after spinning.

### Preparation of Silk mats

The silk mats were prepared as described by Armato^59^ and stored in sterile distilled water. Briefly, Am and Bm mats were prepared separately by degumming to remove the sericin by alkaline method (0.02 M Na_2_CO_3_) and dried in an oven at 50 °C for 3 days^60^. The mats of approximately 1 cm × 1 cm pieces were sterilized in 70% ethanol for 15 min followed by rinsing in sterile phosphate-buffered saline (PBS). For cell culture, the individual mats were placed in 24-well cell culture plates and moistured with culture media before cells were added.

### Biophysical Characterizations

#### Scanning electron microscopy

The surface morphologies of the Calcium oxalates, Am, degummed Am and Bm mats were obtained after gold sputtering using an S-3400N SEM; Hitachi, Japan and photographed at a voltage of 15 kV and room temperature.

#### Energy-dispersive X-ray spectroscopy

The chemical composition of Calcium oxalates, Am, degummed Am and Bm mats were determined by EDX (INCA Penta FET 33, Oxford Instrument UK).

#### Fourier transform infrared spectroscopy

Measurements FTIR spectra of the Calcium oxalates, Am, degummed Am and Bm mats using KBr pellets were obtained on a NICOLET 6700, USA FTIR spectrometer with a spectral resolution from 4000 to 400 cm^−1^.

#### X- Ray Diffraction

To investigate the structure and crystallinity of the samples by XRD using a Philips XPERT-PRO with Cu-Ka radiation (λ1- 5405980 nm). Data was collected from 5 to 70 °C. The voltage and current of the X-ray source were 45 kV and 40 MA respectively, and the step size 2θ 0.001.

### Differential Scanning Calorimeter measurement

DSC thermal scans were carried out on a Universal V4.5A TA instrument at a heating rate of 10 °C min^−1^ in a nitrogen flow 50 mL min^−1^ with the temperature ranging from 30 to 450 °C.

### Thermogravimetric analysis

The changes in physical and chemical properties of materials are measured as a function of increasing temperature using a Universal V4.5A TA instrument with a heating rate of 10 °C min^−1^ up to 800 °C.

### Cell Culture

HaCaT human keratinocyte cells were cultured in Dulbecco’s modified Eagle’s medium supplemented with 10% fetal bovine serum and 1% penicillin/streptomycin at 37 °C in a humidified atmosphere of 5 vol% CO_2_ and 21 vol% O_2_. At approximately 80–90% confluence, the cells were trypsinized for passaging. Mats were sterilized with 70% ethanol and washed with PBS. Samples were treated with collagen solution (concentration: 20 µg mL^−1^) for 20 min and washed with PBS and without collagen served as negative control. The mats were immersed in culture medium overnight before cell seeding. Each mat was placed in a well on a 24-well culture plate. A suspension of keratinocytes (2 × 10^4^ cell mL^−1^) was seeded onto each silk mat, and cells were allowed to attach for 24 h at 37 °C. Cells cultured in a well without any material served as a control.

### Cell morphology and imaging

The LIVE/DEAD Viability/Cytotoxicity Kit (Molecular Probes, Thermo Fisher) was used to observe the viability of keratinocytes within the silk mats constructs. After 24 h of growth, the seeded matrices were fixed in 4% paraformaldehyde for 10 min, followed by membrane permeabilisation by 0.2% Triton X-100 in PBS for 5 min. Mats were washed with sterile PBS and immersed in PBS solution containing 2 mM calcein AM and 1 mM ethidium homodimer and incubated for 30 min. Then, Hoechst nuclear staining was performed for 5 min. Subsequently, the constructs were observed under a Leica DMI 4000B Epi fluorescence microscopy. The cell number was determined at three different locations for each sample using NIH ImageJ (1.49 V) software. At least five samples were tested for each time point and the experiments were repeated thrice.

### Statistical analysis

Data are expressed as the mean ± SD of at least three independent experiments. Statistical significance of differences was evaluated by one-way ANOVA with SPSS 20.0 statistical analysis package followed by Bonferroni’s post-hoc test. p < 0.05 was considered statistically significant.
